# Rapid Simultaneous Quantification of 1-Formyl-2,2-Dimethylhydrazine and Dimethylurea Isomers in Environmental Samples by Supercritical Fluid Chromatography–Tandem Mass Spectrometry

**DOI:** 10.3390/molecules27155025

**Published:** 2022-08-07

**Authors:** Denis V. Ovchinnikov, Sergey A. Vakhrameev, Danil I. Falev, Nikolay V. Ul’yanovskii, Dmitry S. Kosyakov

**Affiliations:** 1 Laboratory of Environmental Analytical Chemistry, Core Facility Center “Arktika”, Northern (Arctic) Federal University, Arkhangelsk 163002, Russia; 2 Federal Center for Integrated Arctic Research, Arkhangelsk 163000, Russia

**Keywords:** formic acid dimethylhydrazide, dimethylurea, rocket fuel, transformation products, supercritical fluid chromatography, tandem mass spectrometry

## Abstract

When released to the environment, the rocket fuel unsymmetrical dimethylhydrazine (UDMH) undergoes oxidative transformations, resulting in the formation of an extremely large number of nitrogen-containing transformation products, including isomeric compounds which are difficult to discriminate by common chromatography techniques. In the present work, supercritical fluid chromatography–tandem mass spectrometry (SFC-MS/MS) was proposed for resolving the problem of fast separation and simultaneous quantification of 1-formyl-2,2-dimethylhydrazine (FADMH) as one of the major UDMH transformation products, and its isomers—1,1-dimethylurea (UDMU) and 1,2-dimethylurea (SDMU). 2-Ethylpyridine stationary phase provided baseline separation of analytes in 1.5 min without the distortion of the chromatographic peaks. Optimization of SFC separation and MS/MS detection conditions allowed for the development of rapid, sensitive, and “green” method for the simultaneous determination of FADMH, UDMU, and SDMU in environmental samples with LOQs of 1–10 µg L^−1^ and linear range covering three orders of magnitude. The method was validated and successfully tested on the real extracts of peaty and sandy soils polluted with rocket fuel and UDMH oxidation products. It was shown that both UDMU and SDMU are formed in noticeable amounts during UDMH oxidation. Despite relatively low toxicity, UDMU can be considered one of the major UDMH transformation products and a potential marker of soil pollution with toxic rocket fuel.

## 1. Introduction

Despite the increasingly active use of environmentally friendly types of rocket propellants (kerosene, methane, and hydrogen in combination with liquid oxygen as an oxidizer), the space programs of different countries still rely on the operation of launch vehicles or booster blocks using toxic unsymmetrical dimethylhydrazine (UDMH) as a fuel [1,2]. When released to the environment, UDMH rapidly undergoes oxidative transformations via radical mechanism resulting in the formation of extremely large number (up to one thousand) of nitrogen-containing transformation products including toxic and carcinogenic compounds [3,4]. To date, several dozen UDMH transformation products have been reliably identified [5,6,7], the most abundant of them are *N*-nitrosodimethylamine (NDMA), formaldehyde dimethylhydrazone, 1,1,4,4-tetramethyltetrazene, *N*,*N*-dimethylformamide, dimethylaminoacetonitrile, 1-methyl-1H-1,2,4-triazole, and 1-formyl-2,2-dimethylhydrazine (formic acid *N*′,*N*′-dimethylhydrazide, FADMH) [8,9,10,11,12,13]. The latter compound has been identified as an UDMH transformation product relatively recently [9], although it is always found in significant amounts in soils and waters contaminated with rocket fuel [12,14,15]. Analyses of UDMH oxidation products in model laboratory experiments and real soil samples by atmospheric pressure ionization high-resolution mass spectrometry [4] showed the presence, in all mass spectra, of an intense signal of the compound C_3_H_8_N_2_O, which can be attributed to FADMH. However, the use of hydrogen/deuterium isotopic exchange mass spectrometry allowed the discrimination of at least three structures with the indicated elemental composition and suggestion of the presence of *N*,*N*-dimethylurea (unsymmetrical dimethylurea, UDMU) among them [3]. This assumption was confirmed by the published data indicating the identification of UDMU by GC-MS [1]. Another but less probable product with the same elemental composition is *N*,*N*′-dimethylurea (symmetrical dimethylurea, SDMU), the discovery of which among UDMH transformation products has not yet been reported in the literature. Since the available information on the possibility of the formation of substantial amounts of UDMU and SDMU along with FADMH during the oxidative transformation of rocket fuel is extremely scarce and requires additional studies, the development of approaches to the simultaneous quantification of these isomeric compounds in complex objects is of great interest. The solution of this problem is complicated by the high polarity and similarity of the physicochemical properties of FADMH, UDMU, and SDMU (Table 1).

In the case of analytes with reactive amino groups, high-performance liquid chromatography (HPLC) is considered a preferred separation technique since it does not require the tedious procedures of preliminary derivatization and matrix change. In combination with mass spectrometry (MS), it has been successfully used for quantification of hydrazines and most of the above-mentioned major UDMH transformation products [17] and made it possible to achieve the limits of FADMH quantification (LOQs) at a level of 0.01–6 μg L^−1^ [11,12]. However, in the case of polar non-ionogenic compounds as FADMH and dimethylurea isomers, the retention on commonly used reversed and ion-exchange stationary phases is relatively weak and separation selectivity is insufficient. Due to the close polarities of such analytes, the use of hydrophilic interaction liquid chromatography also does not allow for the complete separation of FADMH and its isomers and effective elimination of matrix effects.

In our opinion, the most promising method for the simultaneous determination of these compounds is supercritical fluid chromatography–mass spectrometry (SFC-MS) providing separation based on the specific interactions of analytes with polar stationary phase, which is “orthogonal” [18] to a reversed phase HPLC. The low viscosity and high diffusion coefficients of the sub- or supercritical carbon dioxide, which is used as a main component of the mobile phase in SFC, ensure high separation speed and efficiency. Even though there are no published works devoted to the use of SFC or SFC-MS for the determination of UDMH transformation products, SFC has shown itself superior to HPLC in the analysis of isomers [19,20] and various polar compounds [21,22,23]. The combination of SFC with tandem mass spectrometry detection (SFC-MS/MS) provides high sensitivity and selectivity of analyses of complex objects and does not require any additional specific equipment [24,25,26]. Currently, this analytical technique is increasingly used in practice and appears to be a promising alternative to HPLC-MS/MS. The present work is aimed at the development of a rapid and sensitive SFC-MS/MS method for the simultaneous determination of FADMH, UDMU, and SDMU in water samples and soil extracts and thus obtaining new knowledge on the rocket fuel transformation processes in the environment.

## 2. Results and Discussion

### 2.1. Mass Spectra of Analytes and Mass Spectrometry Detection

Being isomeric compounds and having the same molecular weight, all analytes give the signals of protonated molecules [M + H]^+^ at *m*/*z* 89 under the conditions of positive ion mode atmospheric pressure ionization. Despite the evidence previously noted for SFC-MS certain gain in ionization efficiency of nitrogen-containing compounds under electrospray (ESI) conditions compared to atmospheric pressure chemical ionization (APCI) [27], in our preliminary tests, both techniques demonstrated the close intensities of [M + H]^+^ signals at high (>1 mL min^−1^) flowrates of the mobile phase. In this situation, an APCI technique was chosen for further method development due to its less susceptibility to matrix effects. Tandem mass spectrometry in multiple reaction monitoring (MRM) mode was used to ensure the high selectivity of analysis considering relatively low retention of analytes and thus the possibility of co-elution with matrix components. The recorded tandem mass spectra (Supplementary Materials Figure S1) demonstrate the difference in the collision induced dissociation (CID) pathways of UDMU and SDMU—the first compound predominantly eliminates NH_3_ from primary amino group (*m*/*z* 89 → 72), while the second one is characterized by an easy loss of methylamine (*m*/*z* 89 → 58). These ion transitions were chosen for quantification purposes. In the case of FADMH, CID results in a cleavage of *N*-*N* bond with the formation of protonated *N*-methylmethanimine (*m*/*z* 44) or simultaneous loss of carbonyl and methyl groups leading to the formation of methyldiazene or formaldehyde hydrazone (*m*/*z* 45) protonated molecules. The intensity ratios of the corresponding peaks in mass spectra strongly depend on the applied collision energy. As a result of automated optimization of the collision energies for both MRM transitions, the product ion with *m*/*z* 45 was chosen as a quantifier. The optimized parameters of MRM detection are summarized in Table 2.

### 2.2. Screening of SFC Stationary Phases and Optimization of Separation Conditions

The key factor affecting the retention and separation of polar analytes is the nature of the stationary phase. At the first stage of the study, silica-based stationary phases with a particle size of 1.7–3 µm (see Section 3.2) differing in the chemistry of the bonded groups were screened: bridged ethylene hybrid bare silica (Acquity BEH), three octadecyl stationary phases (endcapped Titan C18, Acquity HSS C18 with no endcapping, Nucleodur ISIS with cross-linked octadecyl groups), two sorbents with embedded polar non-ionogenic groups (Nucleodur PolarTec with amide linker between silica surface and octadecyl group, Acquity HSS Cyano with cyanopropyl bonding and high silanol activity), and two stationary phases with polar ionogenic groups (Acquity BEH 2-EP with 2-ethylpyridine moiety and Nucleodur NH2-RP with aminopropyl groups). The chromatograms obtained under common SFC conditions (backpressure 130 bar, mobile phase—10% methanol in CO_2_) (Figure S2) showed potential for the rapid separation of analytes and specificity of their retention on different stationary phases. First, the difference in the behavior of FADMH and dimethylurea isomers should be noted. Despite the close polarities (Log*P*) of all analytes (Table 1) and the presence of the same functional groups, the retention of FADMH is much lower compared to UDMU and SDMU. It is also worth noting that extremely strong tailing and even splitting of FADMH peaks obtained for most stationary phases occur. This can be explained by the higher ability of this compound for protonation (acidity constant of conjugated acid is 4 orders of magnitude lower when compared to other analytes) and thus the presence in mobile phase mostly as cation due to acidic conditions in carbon dioxide–methanol mixture containing significant amounts of methylcarbonic acid [28,29]. The strong interactions of both cationic and neutral forms of FADMH with silica surface leads to the above-noted peak distortions observed mostly for the sorbents with most accessible silanol groups—Acquity BEH, HSS Cyano, and HSS C18. In contrast, the similar Nucleodur ISIS octadecyl phase with a well-shielded silica polar surface provides an acceptable FADMH peak shape, while it does not ensure sufficient retention and separation of UDMU and SDMU, for which the contribution of polar interactions with silanols is crucial. This is in a good agreement with our recent observation of polar retention of pentacyclic triterpenoids on octadecyl stationary phases in SFC at low contents (<6%) of methanol in the mobile phase [30]. An interesting fact is an inversion of symmetrical/unsymmetrical dimethylurea isomers elution order on different stationary phases. Bare silica and sorbents capable of hydrogen bonding and ion exchange (BEH 2-EP, NH2-RP, and PolarTec) provide stronger retention of SDMU, while four other stationary phases (octadecyl and cyanopropyl) are characterized by reversed elution order. While a noticeable contribution of nonpolar retention can be a reason in the case of C18 sorbents, a satisfactory explanation of this phenomenon for cyanopropyl stationary phase has not been found. Among all tested stationary phases, Acquity BEH 2-EP demonstrated best peak shapes and baseline separation of all analytes, although retention factors were relatively low. Combination of hydrogen bond donor and acceptor properties with the capability of π-π interactions allows us to consider this sorbent as universal stationary phase for SFC separations of many polar analytes. Moreover, due to the superior peak shapes even without using mobile phase additives BEH 2-EP is known as the best choice in the analyses of various basic nitrogen-containing compounds [31,32]. Thus, further method development and optimization steps in our work were carried out using Acquity BEH 2-EP chromatographic column.

It has been found that the introduction of formic acid (0.1%, *v*/*v*), ammonium formate (10 mM) and water (5% *v*/*v*) as mobile phase additives (dynamic modifiers), regulating pH and the availability of silanol groups of the sorbent, did not have a significant effect on the retention of analytes, the shape of the chromatographic peaks, and the separation selectivity. Since APCI, unlike ESI, is not sensitive to the analytes protolytic equilibria in the mobile phase, the addition of formic acid did not affect the ionization efficiency and, therefore, the sensitivity of mass spectrometric detection. In this regard, neat methanol was recommended for use as a co-solvent for carbon dioxide. With an increase in the methanol content the retention times (t_R_) of all analytes expectedly decreased due to the polar retention mechanism. This factor led to a simultaneous decrease in separation selectivity. At the same time, a substantial improvement in the chromatographic peak shapes and widths was observed (Supplementary Materials Table S1). The methanol content of 10% (*v*/*v*) was found to be optimal and allowed the separation of analytes with selectivity (α) and resolution (*R*) of >1.5 and 2.0, respectively (Table S1).

Temperature and backpressure usually do not have a significant effect on SFC separations with polar stationary phases and are often considered as secondary parameters when optimizing a chromatographic method [33]. Indeed, we noted some improvement in the chromatographic peak shapes (especially for FADMH) and a slight decrease in retention times with an increase in backpressure, which can be partially compensated by an increase in temperature. Based on these considerations, the operating backpressure of 190 bar and column temperature of 55 °C close to the maximum possible (in terms of the SFC system performance and ensuring the lifetime of the chromatographic column) values were chosen as optimal.

**Figure 1:**
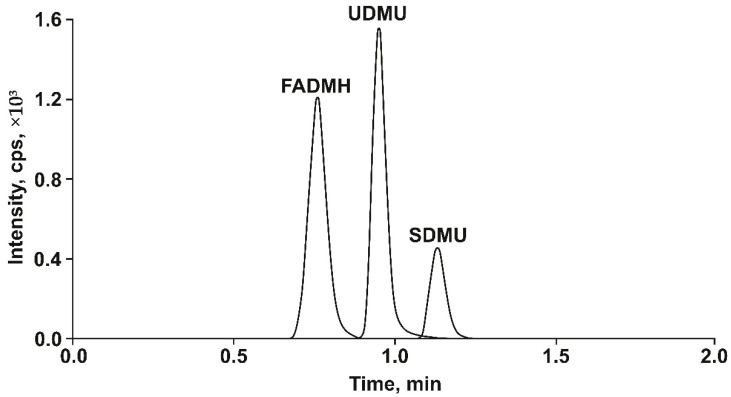
The SFC-MS/MS chromatogram of the model mixture of analytes (3 μg mL^−1^ of FADMH, 0.5 μg mL^−1^ of UDMU and SDMU) obtained under the optimized conditions.

Summarizing the above, the following analysis conditions can be recommended: stationary phase—Acquity UPC2 BEH 2-EP; flow rate—1.30 mL min^−1^; methanol content in the mobile phase—10% (*v*/*v*); temperature—55 °C; backpressure—190 bar. The chromatogram of the model mixture of analytes (Figure 1) obtained under the indicated conditions demonstrates the correct peak shapes and baseline separation of analytes in the isocratic elution mode with an analysis time of 1.5 min.

### 2.3. Validation of the Developed Method

The attained values of instrumental limits of detection (LODs) and limits of quantification (LOQs) (Table 3) were typical for HPLC-MS/MS technique and fell within the ranges of 0.4–3 and 1.3–10 µg L^−1^, respectively. The analyses of calibration solutions of analytes and construction of calibration dependences of peak areas (y) on concentration (x) in the form y = *a*x showed good linearity (*R*^2^ > 0.999) in the concentration range covering three orders of magnitude.

In the case of non-aqueous samples (e.g., acetonitrile extracts of soils [34]) the LODs/LOCs can be further reduced by an increase in the sample injection volume up to 5 µL or even more which is allowed by the used chromatographic column. However, it is strongly not recommended for aqueous samples due to the observed smearing of the chromatographic peaks when injection volumes >2 µL are used. This effect is associated with competition of water and analytes for sorption centers of the stationary phase.

Inter-day and intra-day assays carried out on the model solutions at three concentration levels (from ~LOQ) within an entire linear range (Supplementary Materials Table S2) demonstrated high precision (RSD < 15%) of the developed method at the lowest concentrations of analytes. Moreover, at the level of ≥10, LOQ RSD values were below 4% even in inter-day precision test.

An accuracy of the method was evaluated by spike recovery test using real samples of river water (sample 1) and acetonitrile extract of peat bog soil (sample 2) with very complex matrix (high content of natural organic matter), not containing the studied analytes. The obtained spike recoveries (Table 4) were in the range of 90–115%, including those measured at the LOQ level. Thus, taking into account the high precision of the method, the matrix effects can be considered insignificant even in the case of peat bog water.

The high robustness of the method is due to the use of isocratic elution ensuring the high analysis reproducibility. It was confirmed in the analyses of a great number of real samples without noticeable change in retention times or selectivity loss.

The comparison of the developed method with those described in the literature for single analytes and based on GC [14,34,35] or HPLC [12] separations showed sensitivity similar to GC-MS or HPLC-MS and at least one order of magnitude gain in LOD for SDMU when compared with the GC-NPD method provided by the U.S. Environmental Protection Agency (EPA) [36]. It is worth noting that the SFC-MS/MS method, unlike those mentioned, is distinguished with exceptional rapidity (1.5 min), low consumption of organic solvent, and low cost of the mobile phase.

### 2.4. Analyses of Real Samples

**Figure 2:**
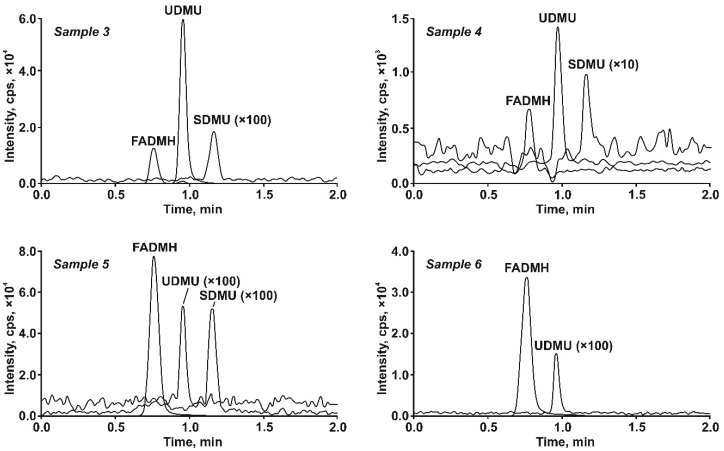
SFC-MS/MS chromatograms of real samples containing UDMH transformation products.

The developed method was successfully tested in the analyses of four real samples containing UDMH transformation products—acetonitrile extracts of soils polluted with rocket fuel and taken from the site of accidental crush of the launch vehicle near Baikonur spaceport (sample 3) and from the landing site of the launch vehicle’s burned-out first stage in northern Russia (sample 4), as well as aqueous solutions of UDMH treated with ozone (sample 5) and hydrogen peroxide in the presence of Cu^2+^ ions as catalyst (sample 6). The recorded chromatograms (Figure 2) revealed the presence of all analytes with concentrations >LOQ. An exception is sample 6, in which SDMU was not detected. As expected, FADMH predominated in all samples (Table 5) and was found at the levels reaching 200 mg L^−1^ (sample 5). This value corresponds to 20% of the initial UDMH content and allows the consideration of FADMH as a main primary transformation product formed under action of ozone.

Another advanced oxidation process, based on the UDMH catalytic treatment with hydrogen peroxide, also provided the conversion of UDMH mainly to FADMH (10% of initial UDMH after 24 h reaction). The results of the model experiments on UDMH oxidation were in a good agreement with the high contents of FADMH found in the soil extracts. For example, sandy soil (sample 3) polluted with rocket fuel and subjected to reagent treatment with hydrogen peroxide contained 190 mg kg^−1^ of this transformation product. Peat bog soil sample contained lower amounts of FADMH; however, the measured content (4.2 mg kg^−1^) was also significant considering the smaller scale of fuel spill and the known ability of peat to strongly bind UDMH and thus prevent its oxidative transformations [15].

Of great interest is the data obtained for the first time on the levels of dimethylurea isomers in polluted soils and UDMH aqueous oxidation products. In model experiments with ozone and hydrogen peroxide as oxidants (samples 5 and 6), the concentrations of UDMU and SDMU were about three orders of magnitude lower compared to FADMH, while in soil samples 3 and 4 the ratios FADMH/UDMU were only 1.9 and 4.4, respectively. In contrast, SDMU was found in less amounts in comparison with those detected in sample 5. It is worth noting that in the peat bog soil sample, UDMU and SDMU contents differed by less than four times. This means that the slow transformation of UDMH in soils provides conditions for the formation of significant amounts of UDMU, which can be considered as one of the major UDMH transformation products and an important marker of rocket fuel contamination, regardless of soil type. Another unexpected result is the formation of SDMU despite the absence of the dimethylamine group in its structure. This once again confirms the hypothesis of the radical nature of UDMH oxidative transformations, accompanied by the transfer of methyl radicals [3].

Given the prominent place of dimethylurea, along with FADMH, among UDMH transformation products, it is of considerable interest to evaluate and compare the toxicity of these isomeric compounds. The in silico prediction involving models based on quantitative structure–activity/toxicity relationships (QSAR/QSTR) revealed relatively low (Category IV) and comparable acute toxicity of all three analytes (Supplementary Materials Table S3). The similar pattern was observed for aquatic toxicity; however, it should be noted that the estimated LC_50_ values for SDMU turned out to be much lower (up to one order of magnitude) than those for FADMH and UDMU. The situation is different in the case of mutagenicity. The predicted probability of positive Ames test is quite high (0.51) for FADMH, while both dimethylurea isomers had this value below 0.20.

## 3. Materials and Methods

### 3.1. Analytes, Reagents and Materials

1,1-Dimethylurea and 1,3-dimethylurea were purchased from Alfa Aesar (Karlsruhe, Germany) and had a purity of ≥97%. 1-formyl-2,2-dimethylhydrazine was synthesized from UDMH (>98%, Sigma-Aldrich, Steinheim, Germany) and ethyl formate (97%, Sigma-Aldrich, Germany) by the known procedure [37].

In SFC-MS/MS analyses, carbon dioxide (99.99%) and HPLC gradient grade methanol purchased from Cryogen (Aramil, Russia) and Khimmed (Moscow, Russia), respectively, formic acid (>98%, Sigma-Aldrich, St. Louis, MO, USA), 10 M aqueous solution of ammonium formate (Sigma-Aldrich, St. Louis, MO, USA), and ultrapure Type I Milli-Q water were used as components of mobile phase and dynamic modifiers.

HPLC gradient grade acetonitrile (0 grade, Cryochrom, St.-Petersburg, Russia), barium hydroxide (pure, Panreac, Barcelona, Spain), sulfuric acid, and “chem. pure” grade isopropanol (Komponent-Reaktiv, Moscow, Russia) were used in the soil extraction procedure and for the preparation of analyte solutions. High purity 30–35% aqueous solutions of hydrogen peroxide and copper sulfate (>98%) purchased from Neva-Reaktiv (St.-Petersburg, Russia) were used in the procedure of UDMH oxidation.

The stock solutions of individual analytes in methanol with concentration of 2 mg mL^−1^ were prepared from accurately weighed portions and stored in a freezer at a temperature of −20 °C. Working and calibration solutions were prepared immediately before the experiments by consecutive dilutions of the mixture of stock solutions with isopropanol.

### 3.2. Real Objects and Sample Preparation

Two real samples, which were not contaminated with rocket fuel and thus did not contain the studied analytes, were used in the method validation procedure: 

Sample 1. River water taken from the mouth of the Northern Dvina River. Salinity—140 mg L^−1^; dissolved organic carbon content—13 mg L^−1^.

Sample 2. Acetonitrile extract of peat bog soil, obtained by pressurized liquid extraction (PLE). The sample was taken in the northeast of the Arkhangelsk region (Russia), where most of the fall sites of launch vehicle first stages launched from the Plesetsk cosmodrome are located. 

The approbation of the developed SFC-MS/MS method on real objects was carried out using two samples of soil (PLE extracts), polluted with rocket fuel, and two samples of UDMH aqueous solutions treated with oxidative reagents:

Sample 3. Acetonitrile PLE extract of sandy grey desert soil, taken from the site of the Proton launch vehicle accidental crash near Baikonur cosmodrome (Kazakhstan) in 2013 after on-site detoxification with oxidative reagent based on hydrogen peroxide and iron complexonate according to current regulations [38].

Sample 4. Acetonitrile PLE extract of peat bog soil taken at the fall site of a launch vehicle first stage in the northeast of the Arkhangelsk region (Russia). The moisture content was 89% and organic matter content was 98% (recalculated to oven-dry sample).

Sample 5. An aqueous solution of 1,1-dimethylhydrazine with an initial concentration of 1000 mg L^−1^ subjected to ozone treatment. The oxidizing agent (200 mg h^−1^) was passed with air flow through UDMH solution (10 mL) for 20 min. Ozone was produced from dry air using Enaly 500AF portable ozone generator (Shanghai, China).

Sample 6. An aqueous solution of 1,1-dimethylhydrazine with an initial concentration of 1000 mg L^−1^ after hydrogen peroxide treatment. In total, 20 µL of 30% H_2_O_2_ solution was added to 10 mL of UDMH solution. To accelerate the transformation processes, an addition of copper sulfate as a catalyst was used; the concentration of copper ions in the reaction mixture was 1 mg L^−1^.

PLE of all soil samples with acetonitrile was carried out using an ASE-350 (Dionex, Sunnyvale, CA, USA) accelerated solvent extraction system in nitrogen atmosphere according to the earlier developed procedure [33] briefly described below.

In the case of sandy soil, a thoroughly averaged sample weighing 1.0 g (dry matter) was placed into a 5 mL stainless steel extraction cell. Extraction was carried out at a pressure of 100 bar and a temperature of 100 °C in two 10 min cycles. Between cycles and at the end of the extraction, rinsing with a fresh portions of the solvent (60% of the cell volume) was used. The resulting volume of the obtained extract was ~30 mL.

In the case of peat, a sample weighing 5.0 g (0.55 g of dry matter) was thoroughly mixed with 1.25 g of barium hydroxide and placed in a 10 mL stainless steel extraction cell. Acetonitrile containing 10% of water was used as an extractant. The same extraction conditions and time program used for the sandy soil were used. The resulting volume of the obtained extract was ~50 mL. To neutralize the strongly alkaline medium and remove dissolved barium hydroxide, 1 M sulfuric acid was added to the extract until pH reached 3–5. The barium sulfate precipitate was removed by centrifugation.

Before analysis, samples 3, 5, and 6 were diluted with isopropanol 100-fold and sample 4—10-fold. After filtration through 0.22 µm nylon membrane filter, samples were injected into SFC-MS/MS system.

### 3.3. Supercritical Fluid Chromatography–Tandem Mass Spectrometry

Chromatographic separation was carried out on a Waters Acquity UPC^2^ (Milford, MA, USA) SFC system consisted of two chromatographic pumps for carbon dioxide and co-solvent, autosampler, column thermostat, and back-pressure regulator (BPR). Make-up solvent (methanol) flow was introduced through tee connector after BPR using additional external chromatographic pump Ultimate 3000 RS (Thermo Fisher Scientific, Waltham, MA, USA). A 150 cm PEEK capillary with an internal diameter of 0.075 mm was used as an interface between the SFC system and mass spectrometer.

The following chromatographic columns were tested in stationary phase screening: Acquity UPC2 BEH, 150 × 3.0 mm, 1.7 µm (Waters, Milford, MA, USA); Acquity HSS Cyano, 150 × 3.0 mm, 1.8 µm (Waters, Milford, MA, USA); Acquity UPC2 BEH 2-EP, 150 × 3.0 mm, 1.7 µm (Waters, Milford, MA, USA); Nucleodur NH2-RP, 125 × 2.0 mm, 3.0 µm (Macherey-Nagel, Duren, Germany); Titan C18, 100 × 2.1 mm, 1.9 µm (Supelco, Bellefonte, PA, USA); Acquity UPC2 HSS C18 SB, 150 × 3.0 mm, 1.8 µm (Waters, Milford, MA, USA); Nucleodur PolarTec, 150 × 2.0 mm, 1.8 µm (Macherey-Nagel, Duren, Germany); Nucleodur C18 Isis, 150 × 2.0 mm, 1.8 µm (Macherey-Nagel, Duren, Germany).

Experiments on stationary phase screening were carried out under the following conditions: mobile phase—carbon dioxide containing 10% methanol; flow rate—1.3 mL min^−1^; column temperature—25 °C; back pressure—130 bar; injection volume—2.0 µL; and make-up solvent flow rate—0.10 mL min^−1^. The void volume of the chromatographic system for calculating retention factors (k) was determined from the first perturbation of the baseline. 

Mass spectrometry detection was performed using an AB Sciex 3200 QTrap triple quadrupole tandem mass spectrometer (Concord, ON, Canada) equipped with Turbo-V ion source with atmospheric pressure chemical ionization (APCI) probe. The positive ion mode (APCI+) was used with further ion source parameters: corona needle current—4 µA; source temperature—300 °C; curtain; nebulizing and drying gas pressure—20; and 50 and 30 psi, respectively.

### 3.4. Method Validation

The LODs and LOQs of the analytes were calculated using signal-to-noise ratio (S/N) criteria of 3 and 10, respectively, and then refined in the analysis of solutions with concentrations close to LOQ. The intra-day precision (RSD) was estimated at three levels in a series of consecutive chromatographic analyses of the standard solutions (n = 10). The inter-day precision was determined in the same manner within 48 h (n = 20). The matrix effect and accuracy of analyte quantification were estimated by the spike recovery test at three concentration levels, known amounts of analytes were introduced into river water sample and peaty soil extract, followed by SFC-MS/MS analyses in three replicates.

### 3.5. In Silico Toxicity Prediction

Acute toxicity (mouse, rats) and toxicity towards two aquatic organisms, fathead minnow (*P. promelas*) and water flea (*D. magna*), as well as mutagenicity (probability of positive Ames test) were predicted with the ACD/Labs Percepta software v. 2021.1.3 (Advanced Chemistry Development, Inc., Toronto, ON, Canada) using quantitative structure–activity/toxicity relationship (QSAR/QSTR) algorithms.

## 4. Conclusions

A rapid, highly sensitive, and “green” method for the simultaneous determination of the three isomeric products (1-formyl-2,2-dimethylhydrazine, 1,3-dimethylurea, and 1,1-dimethylurea) of toxic rocket fuel transformations by supercritical fluid chromatography–tandem mass spectrometry was developed, validated, and successfully tested on real objects. The baseline separation of polar analytes was achieved on a 2-ethylpyridinium stationary phase using carbon dioxide with the addition of methanol (10%) as a mobile phase in isocratic elution mode. The attained detection limits are in the range of 0.4–3.0 µg L^−1^, while the linear concentration range covers three orders of magnitude. The method is distinguished by exceptionally short analysis time (1.5 min), low consumption of organic solvents, and low operational cost. Its application to the study of peaty and sandy soils polluted with rocket fuel as well as complex mixtures of 1,1-dimethylhydrazine oxidation products for the first time allowed the estimation of the levels of two dimethylurea isomers and confirmation of their formation in polluted soils. Despite relatively low toxicity, 1,1-dimethylurea can be considered one of the major 1,1-dimethylhydrazine transformation products and a potential marker of soil pollution with toxic rocket fuel due to its high content in soil samples.

## Figures and Tables

**Table 1:** List of analytes and their physicochemical properties.

Analyte	CAS Number	Structural Formula	Molecular Weight, Da	pKa *	Log*P*
1-formyl-2,2-dimethylhydrazine (FADMH)	3298-49-5	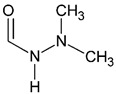	88.1	3.5 ± 0.7 **	−0.81 ± 0.53 **
*N*,*N*-dimethylurea (UDMU)	598-94-7	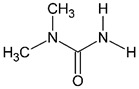	88.1	−0.2 ± 0.7 **	−1.28 ± 0.54 **
*N*,*N*′-dimethylurea (SDMU)	96-31-1	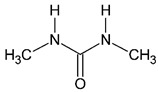	88.1	−0.6 ± 0.7 **	−1.02 ± 0.30 **

* For the protonated form. ** The values predicted in silico by ACD/Labs Percepta platform software [16].

**Table 2:** Detection parameters in the multiple reaction monitoring mode.

Analyte	Precursor Ion, *m*/*z*	Product Ion, *m*/*z*	Declustering Potential, V	Collision Energy, eV
FADMH	89	45 (71 *)	20	20
UDMU	89	72 (46 *)	30	20
SDMU	89	58 (44 *)	30	30

* Qualifier ion.

**Table 3:** The key specifications of the developed SFC-MS/MS method.

Analyte	*a*	*R* ^2^	Linear Range, µg L^−1^	LOD, µg L^−1^	LOQ, µg L^−1^
FADMH	170	0.9998	LOQ-6250	3.0	10
UDMU	1200	0.9995	LOQ-1000	0.4	1.3
SDMU	360	0.9995	LOQ-1000	0.5	1.7

**Table 4:** Accuracy of the method determined by spike recovery test on real samples of river and beat bog water (n = 3, *p* = 0.95).

Analyte	Spiked, µg L^−1^	Found, µg L^−1^		Accuracy, %	
		Sample 1	Sample 2	Sample 1	Sample 2
FADMH	12.5	12.6 ± 1.3	14.3 ± 2.4	101 ± 11	114 ± 17
	310	314 ± 6	320 ± 24	100 ± 3	102 ± 8
	2500	2560 ± 60	2420 ± 70	102 ± 2	97 ± 3
UDMU	2.0	2.2 ± 0.3	2.3 ± 0.3	110 ± 16	115 ± 13
	50	51 ± 2	56 ± 3	102 ± 4	112 ± 5
	400	407 ± 20	410 ± 30	102 ± 5	103 ± 7
SDMU	2.0	2.0 ± 0.2	1.8 ± 0.3	100 ± 10	90 ± 17
	50	49 ± 3	48 ± 4	98 ± 6	96 ± 8
	400	390 ± 15	375 ± 40	98 ± 4	94 ± 11

**Table 5:** Measured concentrations (contents) of analytes in real samples of UDMH transformation products.

Sample	FADMH	UDMU	SDMU
		**Content, mg kg^−1^**	
3	190 ± 15	99 ± 3	1.2 ± 0.1
4	4.2 ± 0.6	0.95 ± 0.09	0.27 ± 0.03
	**Concentration, mg L^−1^**		
5	200 ± 6	0.11 ± 0.04	0.53 ± 0.07
6	105 ± 6	0.36 ± 0.05	<LOQ

## Data Availability

Not applicable.

## Supplementary Materials

The following supporting information can be downloaded at: https://www.mdpi.com/article/10.3390/molecules27155025/s1, Figure S1: Tandem mass spectra of FADMH, UDMU, and SDMU. Collision energy—30 eV with spread of 20 eV; Figure S2: SFC-MS/MS chromatograms of the model mixture of analytes obtained for different stationary phases (Acquity BEH, Titan C18, Acquity HSS C18, Nucleodur ISIS, Nucleodur PolarTec, Acquity HSS Cyano, Acquity BEH 2-EP, Nucleodur NH2-RP). Analysis conditions: mobile phase—carbon dioxide with 10% of methanol; flowrate—1.3 mL min^−1^; column temperature—25 °C; back pressure—130 bar; injection volume—2.0 µL; Table S1: Effect of SFC conditions on the parameters of chromatographic separation of analytes (k—retention factor; W_1/2_—peak width at half-height; α—selectivity; R—resolution); Table S2: The intra-day and inter-day precision of the developed approach; Table S3: In silico predicted toxicity and mutagenicity of analytes.

## Sample Availability

Samples are not available from the authors.
