# Supplementary material for: Rapid Simultaneous Quantification of 1-Formyl-2,2-Dimethylhydrazine and Dimethylurea Isomers in Environmental Samples by Supercritical Fluid Chromatography–Tandem Mass Spectrometry
Source: Molecules. 2022 Aug 7;27(15):5025. doi: 10.3390/molecules27155025 (PMC9370278; doi:10.3390/molecules27155025)
Supplement: Supplementary file 1 [file molecules-27-05025-s001.zip › molecules-1856589-supplementary.pdf]

**Supplementary material**

# Rapid Simultaneous Quantification of 1-Formyl-2,2-Dimethylhydrazine and Dimethylurea Isomers in Environmental Samples by Supercritical Fluid Chromatography - Tandem Mass Spectrometry

Denis V. Ovchinnikov[1], Sergey A. Vakhrameev[1], Danil I. Falev[1], Nikolay V. Ul'yanovskii[1,2,*] and Dmitry S. Kosyakov[1*]

[1] Laboratory of Environmental Analytical Chemistry, Core Facility Center "Arktika", Northern (Arctic) Federal University, Arkhangelsk, Russia
[2] Federal Center for Integrated Arctic Research, Arkhangelsk, 163000, Russia
* Correspondence: d.kosyakov@narfu.ru (D.S.K.); n.ulyanovsky@narfu.ru (N.V.U.)

**Contents**

**Table S1.** Effect of SFC conditions on the parameters of chromatographic separation of analytes (k – retention factor, $W_{1/2}$ – peak width at half-height, $\alpha$ – selectivity, R – resolution).

**Table S2.** The intra-day and inter-day precision of the developed approach

**Table S3.** In-silico predicted toxicity and mutagenicity of analytes

**Figure S1.** Tandem mass spectra of FADMH, UDMU, and SDMU. Collision energy – 30 eV with spread of 20 eV.

**Figure S2.** SFC-MS/MS chromatograms of the model mixture of analytes obtained on different stationary phases (Acquity BEH, Titan C18, Acquity HSS C18, Nucleodur ISIS, Nucleodur PolarTec, Acquity HSS Cyano, Acquity BEH 2-EP, Nucleodur NH2-RP). Analysis conditions: mobile phase – carbon dioxide with 10% of methanol, flowrate – 1.3 mL $min^{-1}$, column temperature – 25°C, back pressure – 130 bar, injection volume – 2.0 μL.

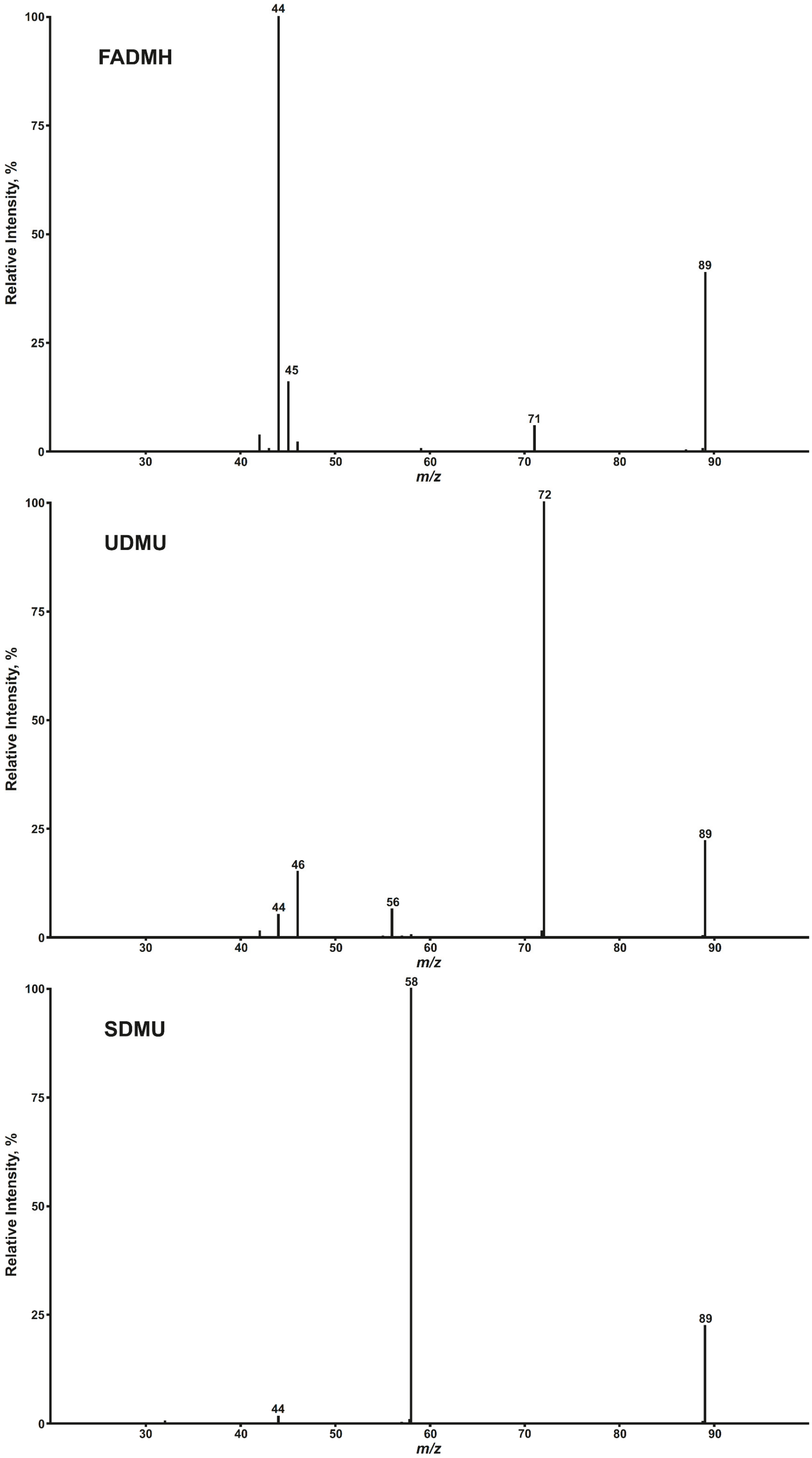


**Figure S1.** Tandem mass spectra of FADMH, UDMU, and SDMU. Collision energy – 30 eV with spread of 20 eV.

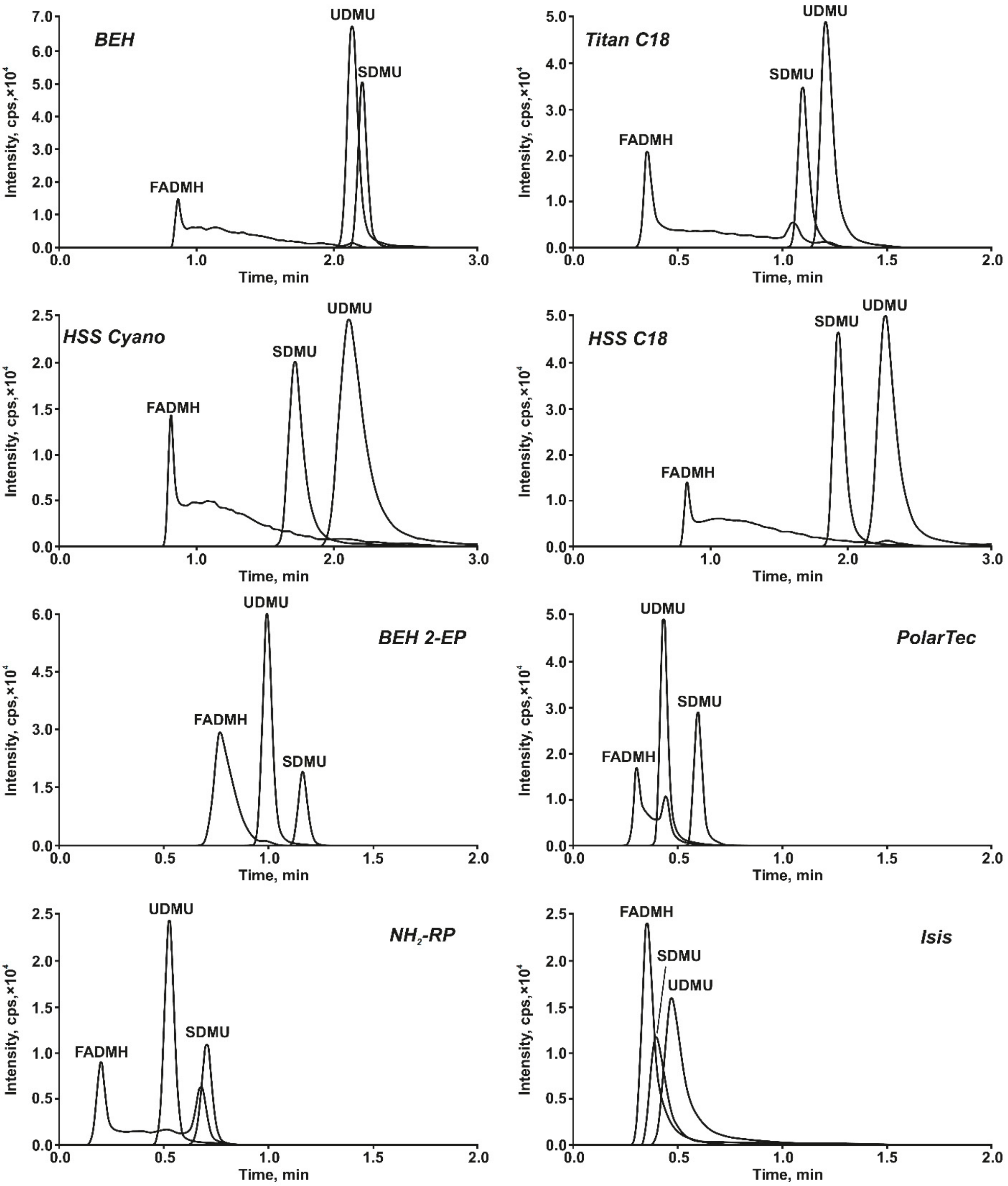


**Figure S2.** SFC-MS/MS chromatograms of the model mixture of analytes obtained on different stationary phases (Acquity BEH, Titan C18, Acquity HSS C18, Nucleodur ISIS, Nucleodur PolarTec, Acquity HSS Cyano, Acquity BEH 2-EP, Nucleodur NH2-RP). Analysis conditions: mobile phase – carbon dioxide with 10% of methanol, flowrate – 1.3 mL min$^{-1}$, column temperature – 25°C, back pressure – 130 bar, injection volume – 2.0 μL.

**Table S1.** Effect of SFC conditions on the parameters of chromatographic separation of analytes (k – retention factor, $W_{1/2}$ – peak width at half-height, $\alpha$ – selectivity, R – resolution).

| Parameter | Value | k | | | $W_{1/2}$ | | | $\alpha$ | | R | |
|---|---|---|---|---|---|---|---|---|---|---|---|
| | | FADMH | UDMU | SDMU | FADMH | UDMU | SDMU | FADMH / UDMU | UDMU / SDMU | FADMH / UDMU | UDMU / SDMU |
| Content of MeOH (% v/v) at 25°C and 130 bar | 5 | 0.43 | 1.59 | 2.95 | 0.11 | 0.07 | 0.09 | 3.70 | 1.86 | 4.79 | 6.34 |
| | 7 | 0.33 | 1.00 | 1.73 | 0.09 | 0.05 | 0.06 | 3.00 | 1.73 | 3.54 | 4.93 |
| | 10 | 0.24 | 0.62 | 0.95 | 0.07 | 0.05 | 0.05 | 2.60 | 1.54 | 2.36 | 2.48 |
| | 12 | 0.19 | 0.48 | 0.70 | 0.06 | 0.05 | 0.05 | 2.50 | 1.47 | 1.93 | 1.65 |
| | 15 | 0.14 | 0.33 | 0.46 | 0.06 | 0.05 | 0.05 | 2.33 | 1.38 | 1.29 | 0.94 |
| T (°C) at 10% of MeOH and 130 bar | 10 | 0.22 | 0.57 | 0.83 | 0.09 | 0.05 | 0.05 | 2.57 | 1.44 | 1.85 | 1.89 |
| | 25 | 0.24 | 0.59 | 0.86 | 0.10 | 0.05 | 0.05 | 2.47 | 1.46 | 1.73 | 2.01 |
| | 40 | 0.24 | 0.59 | 0.89 | 0.09 | 0.05 | 0.05 | 2.47 | 1.51 | 1.85 | 2.24 |
| | 55 | 0.24 | 0.60 | 0.94 | 0.07 | 0.05 | 0.05 | 2.53 | 1.55 | 2.26 | 2.48 |
| P (bar) at 10% of MeOH and 55°C | 110 | 0.25 | 0.65 | 1.02 | 0.08 | 0.05 | 0.05 | 2.56 | 1.56 | 2.27 | 2.71 |
| | 130 | 0.24 | 0.60 | 0.95 | 0.07 | 0.05 | 0.05 | 2.53 | 1.58 | 2.26 | 2.60 |
| | 150 | 0.22 | 0.57 | 0.90 | 0.07 | 0.05 | 0.05 | 2.57 | 1.58 | 2.16 | 2.48 |
| | 170 | 0.21 | 0.54 | 0.86 | 0.07 | 0.05 | 0.05 | 2.62 | 1.59 | 2.07 | 2.36 |
| | 190 | 0.19 | 0.51 | 0.83 | 0.06 | 0.05 | 0.05 | 2.67 | 1.63 | 2.15 | 2.36 |

**Table S2.** The intra-day and inter-day precision of the developed approach

| Analyte | C, μg $L^{-1}$ | Intra-day | | Inter-day | |
|---|---|---|---|---|---|
| | | RSD, % | Accuracy, % | RSD, % | Accuracy, % |
| FADMH | 12.5 | 14 | 103 | 14 | 105 |
| | 125 | 2 | 98 | 2 | 98 |
| | 3125 | 2 | 99 | 3 | 97 |
| 1,1-DMU | 2.00 | 14 | 102 | 15 | 110 |
| | 20.0 | 2 | 94 | 4 | 97 |
| | 500 | 2 | 96 | 3 | 95 |
| 1,3-DMU | 2.00 | 8 | 100 | 10 | 107 |
| | 20.0 | 3 | 96 | 3 | 98 |
| | 500 | 2 | 96 | 2 | 94 |

**Table S3.** In-silico predicted toxicity and mutagenicity of analytes

| Properties | Compound | | |
|---|---|---|---|
| | FADMH | UDMU | SDMU |
| Acute toxicity, $LD_{50}$, mg $kg^{-1}$ | | | |
| Mouse/Intraperitoneal | 500 | 410 | 410 |
| Mouse/Oral | 2200 | 1100 | 1300 |
| Rat/Intraperitoneal | 500 | 720 | 420 |
| Rat/Oral | 2100 | 1300 | 1100 |
| Aquatic toxicity, $LC_{50}$, mg $L^{-1}$ | | | |
| Pimephales promelas | 280 | 460 | 19 |
| Daphnia magna | 440 | 750 | 240 |
| Mutagenicity | | | |
| Probability of positive Ames test | 0.51 | 0.14 | 0.19 |
